# Human Papillomavirus Vaccination at a Time of Changing Sexual Behavior

**DOI:** 10.3201/eid2201.150791

**Published:** 2016-01

**Authors:** Iacopo Baussano, Fulvio Lazzarato, Marc Brisson, Silvia Franceschi

**Affiliations:** International Agency for Research on Cancer, Lyon, France (I. Baussano, F. Lazzarato, S. Franceschi);; University of Turin, Turin, Italy (F. Lazzarato); University of Piemonte Orientale Avogadro, Novara, Italy (F. Lazzarato);; Centre de Recherche du Centre Hospitalier Universitaire, Québec City, Québec, Canada (M. Brisson);; Université Laval, Québec City (M. Brisson);; Imperial College, London, UK (M. Brisson)

**Keywords:** sexual behavior, human papillomavirus 16, viruses, papillomavirus vaccines, vaccination, immunization, sexual behavior, sexual partners, heterosexuality, epidemiologic transition

## Abstract

Human papillomavirus (HPV) prevalence varies widely worldwide. We used a transmission model to show links between age-specific sexual patterns and HPV vaccination effectiveness. We considered rural India and the United States as examples of 2 heterosexual populations with traditional age-specific sexual behavior and gender-similar age-specific sexual behavior, respectively. We simulated these populations by using age-specific rates of sexual activity and age differences between sexual partners and found that transitions from traditional to gender-similar sexual behavior in women <35 years of age can result in increased (2.6-fold in our study) HPV16 prevalence. Our model shows that reductions in HPV16 prevalence are larger if vaccination occurs in populations before transitions in sexual behavior and that increased risk for HPV infection attributable to transition is preventable by early vaccination. Our study highlights the importance of using time-limited opportunities to introduce HPV vaccination in traditional populations before changes in age-specific sexual patterns occur.

## Abstract 2

Early vaccination may prevent infections in populations undergoing age-specific changes in sexual activity.

Changes in sociocultural norms that regulate sexual behavior have been reshaping the epidemiology of sexually transmitted infections (STIs) in many areas of the world. For example, changes in sexual practices have resulted in an epidemic of syphilis and other STIs in China (*1**,**2*). Data from surveys of sexual behavior show considerable heterogeneity of age-specific sexual patterns by country. For example, average age difference between spouses or cohabiting partners ranged from 15 years in Burkina Faso to 2 years in Australia (*3*) (Technical Appendix Figure 1). In addition, marriage at older ages and sexual debut at earlier ages in women have been observed in high-income countries over the past few decades (*4*) and are also now reported in many low- and middle-income countries (*3*). Furthermore, age differences between sexual partners at onset of sexual activity (*5*) and rate of sexual activity in young persons (*2*) have been shown to influence the age-specific distribution of STIs, such as HIV and syphilis, and have been proposed as determinants of international variations in overall and age-specific prevalence of human papillomavirus (HPV) infection (*6*).

We used a transmission-dynamic model to illustrate how changes in age-specific rates of sexual activity and age difference between sexual partners potentially affect HPV prevalence in heterosexual populations. The model also shows how differences in the timing of HPV vaccination relative to changes in age-specific sexual behavior may influence the effectiveness of HPV vaccination programs.

## Methods

We adapted a previously described dynamic model of HPV infection (*7*) to simulate transmission and clearance of the infection (Technical Appendix). We focused on HPV16 infection because type 16 is the most frequent and most carcinogenic HPV type in all world regions (*8*) and is targeted by HPV vaccines.

### Sexual Behavior and Study Populations

We adapted a model that used 1) age- and gender-specific rates of sexual activity per year and 2) distribution of age differences between sexual partners to simulate 2 heterosexual populations, one with traditional age-specific sexual behavior and the other with gender-similar age-specific sexual behavior. Traditional sexual behavior indicates a population in which genders have different age-specific sexual activity rates and a wide gap in ages (e.g., an average of 5.6 years, as observed in India) of spouses or cohabitating sexual partners. Gender-similar sexual behavior indicates a population in which genders have similar age-specific sexual activity rates and a narrow gap in ages (e.g., an average of 2.1 years, as observed in the United States) of spouses or cohabitating sexual partners. In the population with traditional sexual behavior, studies suggest that sexual activity (i.e., having new sexual partners) among women occurs mostly at young ages (i.e., at marital age), whereas sexual activity among men usually reaches a plateau, typically at postmarital age, and remains consistent throughout their sexually active life (*3**,**4**,**9**–**11*). Conversely, in the population with gender-similar sexual behavior, both men and women concentrate most of their sexual activity at young ages and engage in mainly premarital relationships (*7**,**12**–**15*). The number of new sexual partners for this population peaks at ages <25 years. 

We then stratified the simulated populations into 2 levels of sexual activity (high or low), according to age-specific rates of sexual activity. The number of new sexual partners per year was obtained by calibration and used as a proxy for sexual activity. To factor in differences in sexual activity rates for the 2 populations, we assumed and imposed on the model a set of age-specific relative rates to represent observed sexual activity patterns for the 2 groups (Technical Appendix Table 1). We varied the average number of new sexual partners per year from 1 to 2 in the calibration phase, in agreement with values reported in studies that modeled HPV or HIV transmission (i.e., 0.29 and 4.0 partners per year) (*16*).

To represent age differences between sexual partners in the 2 populations with age-different and age-similar sexual patterns, we used age differences between spouses or cohabiting partners reported in India in 2005 (mean 5.6 years; 95% CI 0–13 years) (*17*) and in the United States in 2008 (mean 2.1 years; 95% CI –7 to 11 years) (*18*), respectively. On the basis of available data for age at first intercourse (*3*), sexual activity did not start before 14 years of age for either gender in each simulated population, and all persons were considered susceptible to HPV16 infection when they started sexual activity. We kept constant other demographic, behavioral, and biologic parameters for the 2 populations.

### Model Parameterization and Calibration

Using values that we estimated and validated in our previous work with large sets of high-quality data from high-income countries (*7*), we assumed for the model an 80% probability of transmission per sexual partnership for both genders and a 20% probability of developing type-specific immunity after infection clearance (Table). We also assumed that the per-person annual rate of viral clearance decreased 1.3–0.3 person-years for infections that occurred <1 year to >2 years earlier (Table). The simulated populations are open stable (i.e., age-specific mortality rates are balanced by the birth rate) and stratified by single-year age groups (ages 10–70 years).

**Table:** Model parameters related to HPV16 infection, sexual behavior, and vaccine efficacy and values assigned or calibrated*

Parameter	Value	Source
Probability of transmission per sexual partnership, %	80	Assumed
Fraction of immunity after infection clearance, %	20	Assumed
Rate of clearance by duration since infection, person-year		Assumed
<1 y	1.3	
1–2 y	0.8	
>2 y	0.3	
New sexual partners per year, mean		
Heterosexual population with traditional sexual behavior	2.0	Calibrated
Heterosexual population with gender-similar sexual behavior	1.5	Calibrated
Heterosexual population with gender-similar sexual behavior with increased number of partners	2.0†	SA
Heterosexual population with traditional sexual behavior with decreased number of partners	1.5‡	SA
Mixing between classes of sexual activity§	0.7	Calibrated
	0.3	SA
Vaccination efficacy	95%	Assumed
Duration of vaccine protection	Lifelong	Assumed

*Values have been assumed on the basis of previous research (*7*). We calibrated values by fitting model-based projections to data from rural India (*19*) and the United States (*20*). SA indicates that the value was imposed on the model for univariate sensitivity analysis. † We increased the average number of partners from 1.5, the calibrated value, to 2.0 in the population with gender-similar sexual behavior. ‡ We decreased the average number of partners from 2.0, the calibrated value, to 1.5 in the population with traditional behavior.  §”Mixing between classes of sexual activity” is a measure of the tendency for persons with similar levels of sexual activity to form sexual partnerships. It is measured on a scale where fully and randomly assortative (i.e., like-with-like) mixing corresponds to values 0 and 1, respectively. For the sensitivity analysis, we changed the value of assortative mixing by level of sexual activity from 0.7, the calibrated value, to 0.3.

With our model-based projections, we reproduced data from rural India (*19*) and the United States (*20*) as examples of heterosexual populations with gender-different (i.e., traditional) and gender-similar age-specific sexual behaviors (Technical Appendix Figure 2). Age-standardized HPV16 prevalence (3.6%) in rural India is consistent with prevalence found in traditional populations (*19*) and lower than the corresponding prevalence (5.8%) in the United States (*20*). To obtain adequate matching of model-based projections with the age-specific HPV16 prevalences reported from rural India and the United States, we calibrated the average annual number of new sexual partners (Technical Appendix Table 2) and the tendency for persons with similar sexual activity to form sexual partnerships (i.e., assortative mixing by sexual activity) (Table).

### Model-Based Analyses

To show the effects of vaccination in populations with traditional and gender-similar sexual behaviors, we simulated the introduction of vaccination against HPV16 for 11-year-old girls only and for both girls and boys and calculated the percent reduction in HPV16 prevalence attributable to vaccination in the 2 populations with differing age-specific sexual behavior at the postvaccination equilibrium (i.e., 70 years after introduction of the vaccination). We then sought to show how transitioning from traditional to gender-similar age-specific sexual behavior over a 15-year period affects HPV16 prevalence in women 20–34 years of age. The transition from traditional to gender-similar sexual behavior was simulated by assuming a progressive shift towards gender-similar sexual activity rates and reduction of age gap between sexual partners. Finally, we simulated the introduction of HPV vaccination, with and without catch-up vaccination of girls and women >11 years of age, before and after an age-specific sexual behavior transition period. On the basis of previous reports (Technical Appendix), vaccination coverage was set at 70%, vaccine efficacy against HPV16 was set at 95%, and the duration of protective immunity against HPV16 infection was assumed to be lifelong. To assess the sensitivity of our estimates to the calibrated parameters (i.e., the average number of new sexual partners per year and the assortative mixing by sexual activity), we repeated our analyses and imposed on the model different values than those obtained through model calibration. In particular, we decreased the average number of partners by 0.5 in the population with traditional behavior (i.e., from 2.0, the calibrated value, to 1.5) and increased the average number of partners from 1.5, the calibrated value, to 2.0 in the population with gender-similar sexual behavior. Finally we changed the value of assortative mixing by sexual activity from 0.7, the calibrated value, to 0.3 (on a scale where fully and randomly assortative mixing correspond to values of 0 and 1, respectively; Technical Appendix Figure 3).

## Results

**Figure 1:**
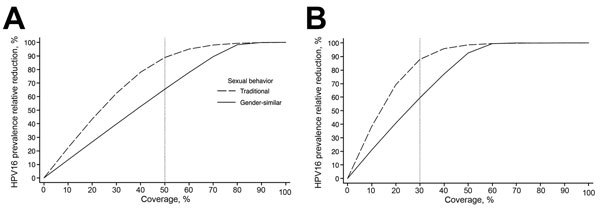
Relative reduction of prevalence of human papillomavirus type 16 at postvaccination equilibrium (i.e., 70 years after the introduction of vaccination) attributable to vaccination among women 20–34 years of age after vaccination of 11-year-old girls or 11-year-old girls and boys, by coverage and a population’s age-related sexual behavior. A) 30% vaccine coverage; B) 50% vaccine coverage. Traditional sexual behavior indicates a population in which genders have different age-specific sexual activity rates and a wide gap in ages (e.g., an average of 5.6 years, as observed in India) of spouses or cohabitating sexual partners. Gender-similar sexual behavior indicates a population in which genders have similar age-specific sexual activity rates and a narrow gap in ages (e.g., an average of 2.1 years, as observed in the United States) of spouses or cohabitating sexual partners.

We used the simulations to compare the percent reduction in HPV16 prevalence attributable to vaccination by coverage level after introduction of a vaccination program (for 11-year-old girls only and for both girls and boys) in a traditional sexual-behavior population and in a population with gender-similar sexual behavior (Figure 1). At the postvaccination equilibrium, the estimated percent reduction in HPV16 prevalence attributable to vaccination is larger in the traditional population than in the population with gender-similar sexual behavior, and differences persist until coverage for girls-only vaccination is at 80% and coverage for gender-neutral vaccination is at 60%. These levels of vaccination coverage are sufficient to eliminate HPV16 infection in the population with gender-similar age-specific sexual behavior. In a girls-only vaccination program, the largest projected difference in reduced prevalence attributable to vaccination for the 2 populations is at ≈50% coverage, which enables a reduced HPV16 prevalence in the traditional sexual-behavior population of 85% compared with a 64% reduction in the population with gender-similar sexual behavior. For vaccination programs targeting girls and boys, the largest difference in reduced prevalence attributable to vaccination is at ≈30% coverage: 83% reduction in HPV16 prevalence for the traditional sexual-behavior population versus 58% reduction for the population with gender-similar sexual behavior.

**Figure 2:**
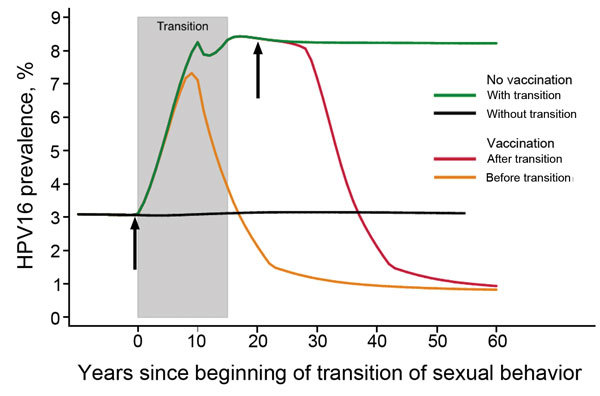
Changes in prevalence of human papillomavirus type 16 among women 20–34 years of age in relation to the number of years since the beginning of a population’s transition from traditional to gender-similar age-related sexual behavior and the introduction of vaccination among 11-year-old girls (with assumption of 70% coverage) before and after transition. Shaded area shows an assumption of a 15-year transition period. Arrows show approximate timing of vaccination occurring before or after a transition has occurred. Traditional sexual behavior indicates a population in which genders have different age-specific sexual activity rates and a wide gap in ages (e.g., an average of 5.6 years, as observed in India) of spouses or cohabitating sexual partners. Gender-similar sexual behavior indicates a population in which genders have similar age-specific sexual activity rates and a narrow gap in ages (e.g., an average of 2.1 years, as observed in the United States) of spouses or cohabitating sexual partners.

We also simulated changes in HPV16 prevalence among women 20–34 years of age in relation to the timing of the transition from traditional to gender-similar sexual behavior and HPV vaccination introduction (11-year-old girls only, 70% coverage; Figure 2). Our model showed that in a no-vaccination scenario, HPV16 prevalence increases from 3% to 8% with transition to gender-similar sexual behavior. The introduction of HPV vaccination before transition to gender-similar sexual behavior halts this increase in ≈10 years and induces a decrease in prevalence to ≈1.5% in 20 years. In 30 years, vaccination reduces HPV16 prevalence to ≈1% at equilibrium. In contrast, if vaccination is introduced after transition to gender-similar sexual behavior, HPV16 prevalence will not reach 1% equilibrium for 40 years after vaccination introduction. Advantages of vaccination in populations before age-specific sexual-behavior transitions occur are reduced potential increases of HPV16 prevalence and earlier effects of vaccination.

To assess the sensitivity of our estimates to the assumed average number of new sexual partners per year, we repeated our simulations by imposing the same average number (1.5 and 2.0) of partners on the population with age-similar sexual partners and on the traditional population with age-different sexual partners (Table; Technical Appendix Figure 4, panels A, B). Decreasing the average number of partners by 0.5 in the population with traditional sexual behavior (i.e., from 2.0 to 1.5) and increasing the average number of partners from 1.5 to 2.0 in the population with gender-similar sexual behavior modified the HPV16 prevalence among women 20–34 years of age by similar magnitudes in both populations: 2.4% (from 3.1% to 0.7%) for the population with traditional sexual behavior and 2.8% (from 8.2% to 11.0%) in the population with gender-similar sexual behavior. Despite the sensitivity of HPV16 prevalence to the average number of new sexual partners per year, the benefit of introducing HPV vaccination before transition was confirmed (Technical Appendix Figure 4). In addition, our findings were robust to the assumption of a more assortative mixing between classes of sexual activity, with prevalence increasing from 3.4% to 7.0% with transition in age-specific sexual behavior (Technical Appendix Figure 3).

## Discussion

We show that the effects of a vaccination program are influenced by a population’s age-specific sexual behavior (i.e., traditional or gender-similar). We also highlight the benefits of implementing HPV vaccination in traditional populations before transition to gender-similar sexual behavior occurs. The earlier that vaccination is established in a population undergoing sexual-behavior transition, the more likely it is that vaccination will be highly effective, even if initial coverage is suboptimal. Sensitivity analyses show that our findings are robust to uncertainties about average number of partners and assortative mixing by sexual activity in the 2 types of sexual behavior patterns.

In our simulated traditional population, the transition to gender-similar sexual behavior entails a 2.6-fold increase, from 3% to 8%, in HPV16 prevalence in women 20–34 years of age. In populations with gender-similar sexual behavior, sexual activity of both men and women peaks at young ages (<30 years of age), and age difference between partners is narrow. As a result, the corresponding peaking sexual activity of young women and men is more likely to enable an efficient and rapid spread of HPV infection among susceptible young persons with multiple new sexual partners per year. Nonoverlapping age-specific peaks of sexual activity and larger age differences between sexual partners, as observed in traditional populations, can decrease the basic viral reproductive number and the spread of HPV infection and increase the herd immunity effect of vaccination programs (*21*). Consequently, vaccination has stronger effects in populations with traditional rather than gender-similar sexual behavior. According to our model, the largest difference in percent reduction in HPV16 prevalence attributable to vaccination is seen if coverage is 50% (for girls-only vaccination) or 30% (for girls-and-boys vaccination). With this level of coverage, the reduction in HPV16 prevalence achievable in women 20–34 years of age is estimated to be ≈80%, compared with ≈60% if vaccination is introduced after a transition to gender-similar sexual behavior. Similarly, according to our projections (Technical Appendix Table 3), a 1-time catch-up campaign is more efficient in a population with traditional sexual behavior than in a population that has transitioned to gender-similar sexual behavior. Ambitious catch-up (i.e., up to age 18 or 25 years) would confer protection on women for whom high-quality cervical cancer screening may not be available (*22*).

Our model shows that early implementation of HPV vaccination attenuates increased risk of HPV infection that accompanies transition to gender-similar sexual behavior. This finding affects the interpretation of studies on the surveillance of HPV vaccination. For example, in the absence of reliable data regarding the sexual behavior of a population and on vaccination coverage, increased HPV prevalence might be erroneously interpreted as a lack of vaccine effectiveness.

Our study has strengths and limitations. One strength is the use of a validated transmission model to represent changes in HPV16 prevalence. Transmission models can capture the dynamics of infection circulation (*23*) in a population and have the distinct advantage of including the effect of herd-immunity attributable to vaccination (*24**,**25*). We derived estimates for the parameters governing the natural history of HPV16 infections from a large cervical cancer screening study conducted in Italy (*26*) and validated the estimates by comparing them with data from a large dataset from Sweden (*27*).

Although cervical cancer reduction is the ultimate aim of vaccination, we chose a viral endpoint rather than cervical disease endpoints to avoid the inclusion of additional uncertainties that would be introduced by other parameters that regulate the progression or regression of HPV infection into precancerous cervical lesions and cancer. Viral endpoints are also the earliest to manifest and offer the opportunity to monitor vaccination programs and the adequacy of our model. We also chose to focus on HPV16 only because information about the natural history of types other than HPV16 and about vaccine efficacy against these types is limited, but data are generally consistent for HPV16. However, use of viral endpoints does not eliminate uncertainties related to the acquisition and clearance of HPV16 and subsequent immunity to the virus (*21*).

To keep our model simple, we accounted for heterogeneity in sexual behavior by stratifying the simulated heterosexual populations into 2 classes of sexual activity and did not account for same-sex or concurrent sexual partnerships (*23*). Ideally, an exhaustive description of exposure to HPV infections should consider the representation of the entire sexual network in which HPV infections are transmitted, but such information is rarely available (*28**,**29*). The method we adopted to represent sexual activity has been extensively used to investigate the epidemiology of STI other than HPV (*23*), and the sexual activity rates we used to account for country- and age-specific HPV curves are consistent with those observed in high- and low-income countries (*7**,**9**–**14**,**16**,**30*).

To calibrate our HPV transmission model, we chose to use data from rural India and the United States as examples of populations with traditional and gender-similar sexual behavior, respectively. Obviously, the classification of sexual behavior into traditional or gender-similar behaviors is an oversimplification, as is our assumption that the age-specific profile of HPV16 prevalence could be sufficiently explained by differences in the number of new sexual partners and age difference between heterosexual partners. Other authors have evoked HPV reactivation as a cause of high HPV prevalence in older women (*31*). In the absence of specific information, we assumed that the increase in premarital sex that characterizes the transition to gender-similar sexual behavior (*3**,**4*) can be accounted for by modulating the number of new sexual partners as a function of age. Likewise, we have assumed that recorded age differences between cohabiting partners or spouses in India and in the United States are representative of age differences between sexual partners in general.

On the basis of results of our model, we find that traditional or gender-similar age-specific sexual behavior can shape age-specific HPV prevalence curves and that a particularly favorable, time-limited window currently exists for the introduction of HPV vaccination in traditional populations in low- and middle-income countries. National and international agencies should seize this opportunity with adequate political commitment, planning, and funding.

**Technical Appendix.** Additional details of methods and results related to catch-up vaccination scenarios and sensitivity analyses.
