# Supplementary material for: Human Papillomavirus Vaccination at a Time of Changing Sexual Behavior
Source: Emerg Infect Dis. 2016 Jan;22(1):18–23. doi: 10.3201/eid2201.150791 (PMC4696692; doi:10.3201/eid2201.150791)
Supplement: Supplementary file 1 — Technical Appendix. Additional details of methods and results related to catch-up vaccination scenarios and sensitivity analyses. [file 15-0791-Techapp-s1.pdf]

Article DOI: http://dx.doi.org/10.3201/eid2201.150791

Human Papillomavirus Vaccination at a Time of Changing Sexual Behavior

Technical Appendix

Additional Details Regarding Methods and Results

Transmission model description

The transmission model used to simulate the transmission and natural history of HPV16 infection and to project the impact of vaccination has been extensively described and validated elsewhere (*3*). Briefly, we developed a partial integro-differential equation model of heterosexually transmitted HPV infection. HPV16 was modeled independently from the other types. The model accounted for the effect of several time scales: calendar, age, and time since infection (as a determinant of infection clearance).

To estimates the values of HPV16 biologic parameters (i.e., probability of infection transmission per sexual partnership, rate of clearance by duration since infection, and fraction of immunity after infection clearance), we separately fitted the model's outputs to the HPV16 and age-specific prevalence curves observed in large population-based studies from Italy and Sweden. For both countries, information on sexual behavior was collected from nationwide population-based surveys and applied to study populations.

We represented the network of sexual partnerships by making some simplifications about contact patterns within the 2 populations: a) all sexual contacts were heterosexual; b) concurrent sexual partnerships were not explicitly accounted for; c) the annual rate of acquisition of new sexual partners varied only by age-group (5-year age-groups ranging between 14 and 75 years) and class of sexual activity; and d) sexual preferences were represented in terms of age and sexual activity assortativeness (i.e., the tendency of persons with similar age and sexual activity to form sexual partnerships).

Finally, the estimation process for parameters was conceived as 2 independent sets of simulations that were blind to the other's outputs and used the same transmission model and methodology. We assessed the validity of estimates by cross- and out-of-sample validation tests.

Model-Based Analyses

Recent data from school- and community-based vaccination programs in medium- and low-income countries show that a 3-dose vaccination coverage of young girls can range between ≈70% and >90% (*4*,*5*). In our model analyses, vaccination coverage was conservatively assumed to be 70% for 11-year-old adolescent girls, with a 95% vaccine efficacy against HPV16 on the basis of reported vaccine trials. Lifelong immunity is the current expected duration of vaccine-induced immunity (*6*).

References

1. United Nations Development Program. Human development reports. Human development index (HDI) [cited 2014 Oct 7]. http://hdr.undp.org/en/statistics/hdi
2. Measure DHS. Demographic and health surveys [cited 2014 Oct 7]. https://dhsprogram.com/Who-We-Are/upload/MEASURE_DHS_Brochure.pdf
3. Baussano I, Elfstrom KM, Lazzarato F, Gillio-Tos A, De Marco L, Carozzi F, et al. Type-specific human papillomavirus biological features: validated model-based estimates. PLoS ONE. 2013;8:e81171. PubMed
4. Binagwaho A, Wagner CM, Gatera M, Karema C, Nutt CT, Ngabo F. Achieving high coverage in Rwanda's human papillomavirus vaccination programme. Bull World Health Organ. 2012;90:623–8. PubMed http://dx.doi.org/10.2471/BLT.11.097253
5. Watson-Jones D, Baisley K, Ponsiano R, Lemme F, Remes P, Ross D. Human papillomavirus vaccination in Tanzanian schoolgirls: cluster-randomized trial comparing 2 vaccine-delivery strategies. J Infect Dis. 2012;206:678–86. PubMed http://dx.doi.org/10.1093/infdis/jis407
6. Lehtinen M, Dillner J. Clinical trials of human papillomavirus vaccines and beyond. Nat Rev Clin Oncol. 2013;10:400–10. PubMed http://dx.doi.org/10.1038/nrclinonc.2013.84
7. Garnett GP, Anderson RM. Balancing sexual partnerships in an age and activity stratified model of HIV transmission in heterosexual populations. IMA J Math Appl Med Biol. 1994;11:161–92. PubMed http://dx.doi.org/10.1093/imammb/11.3.161
8. Elbasha EH, Dasbach EJ, Insinga RP. Model for assessing human papillomavirus vaccination strategies. Emerg Infect Dis. 2007;13:28–41. PubMed
9. Franceschi S, Herrero R, Clifford GM, Snijders PJ, Arslan A, Anh PT, et al.; IARC HPV Prevalence Surveys Study Group. Variations in the age-specific curves of human papillomavirus prevalence in women worldwide. Int J Cancer. 2006;119:2677–84. PubMed http://dx.doi.org/10.1002/ijc.22241

10. Dunne EF, Sternberg M, Markowitz LE, McQuillan G, Swan D, Patel S, et al. Human papillomavirus (HPV) 6, 11, 16, and 18 prevalence among females in the United States—National Health And Nutrition Examination Survey, 2003–2006: opportunity to measure HPV vaccine impact? J Infect Dis. 2011;204:562–5. PubMed http://dx.doi.org/10.1093/infdis/jir342

11. Hertog S. Heterosexual behavior patterns and the spread of HIV/AIDS: the interacting effects of rate of partner change and sexual mixing. Sex Transm Dis. 2007;34:820–8. PubMed

12. Tucker JD, Chen XS, Peeling RW. Syphilis and social upheaval in China. N Engl J Med. 2010;362:1658–61. PubMed

Technical Appendix Table 1. Assumed relative annual rates of new sexual partners by gender, age, and level of sexual activity in populations with traditional or gender-similar age-specific sexual behavior*

	Type of Sexual Behavior		
Characteristic	Traditional		Gender similar
Age	Men	Women	Both genders
<20	2	4	10
20–24	2	8	15
25–29	1	4	3
30–34	1	4	3
35–39	1	2	2
40–44	1	2	2
45–49	1	1	1
≥50	1	1	1
Level of sexual activity†			
Low	1	1	1
High	5	5	5

***Rates were assumed and imposed on our model and are relative because they are multiplicative factors (as relative risks), a method used in the literature *(7,8)* to represent age-specific patterns of rates of sexual activity.

†“High” and” low” are standard denominations for classes of sexual activity and indicate classes of sexual activity with a higher and lower number of partners.

Technical Appendix Table 2. Average annual number of new sexual partners by gender, age, and level of sexual activity in populations with traditional and gender-similar age-specific sexual behavior*

	Traditional				Gender similar	
	Men		Women		Both genders	
	Level of sexual activity					
Age	Low	High	Low	High	Low	High
15	0.59	2.96	0.46	2.28	0.60	3.00
20	1.78	8.89	1.64	8.21	1.98	9.91
25	1.60	8.00	2.46	12.31	2.27	11.35
30	0.89	4.44	1.37	6.84	0.54	2.70
35	0.89	4.44	1.23	6.15	0.50	2.52
40	0.89	4.44	0.68	3.42	0.36	1.80
45	0.89	4.44	0.62	3.08	0.32	1.62
50	0.89	4.44	0.34	1.71	0.18	0.90
55	0.89	4.44	0.34	1.71	0.18	0.90
60	0.89	4.44	0.34	1.71	0.18	0.90

*Reported average annual numbers of new sexual partners were obtained by assuming and imposing on the model a set of relative rates of sexual activity (Technical Appendix Table 1) and allowing the average number of new sexual partners per year to vary between 1–2 in the calibration phase, according to values reported in studies that modeled HPV or HIV transmission (i.e., 0.29 and 4.0 partners per year) (*11*).

Technical Appendix Table 3. Relative reduction in HPV16 prevalence attributable to vaccination, by number of years since HPV vaccine was introduced, age at catch-up vaccination, and sexual behavior*

Years since routine vaccination introduction	Age at catch-up† vaccination	Reduced prevalence for traditional sexual behavior, %			Reduced prevalence for gender-similar sexual behavior, %		
			Catch-up age range			Catch-up age range	
		No catch-up	12–18	12–25	No catch-up	12–18	12–25
10	15	6.8	58.3	65.6	5.6	55.9	58.9
	25	2.2	8.2	25.6	1.3	5.4	16.1
20	15	21.7	65.8	72.0	14.9	58.6	60.9
	25	5.8	10.1	27.7	5.0	8.4	19.1

*Table shows the projected reduction attributable to vaccination (%RAV) in HPV16 prevalence in populations with traditional and gender-similar age-specific sexual behavior after routine vaccination of 11-year-old girls only, with or without an additional 1-time catch-up (assumed coverage is 70% for routine and catch-up vaccination). Two types of catch-up are considered (ages 12–18 and 12–25 years), along with 2 birth cohorts: women vaccinated at age 15 in both catch-up scenarios and women vaccinated at age 25 (only in the 12–25 catch-up scenario). The RAV was moderately larger in a population with a traditional sexual behavior than in a population with a gender-similar sexual behavior for all catch-up strategies investigated. After the first 10 (or 20) years after the introduction of vaccination, RAV of HPV16 prevalence was 66% (or 72%) among women vaccinated at 15 years of age in a population with a traditional age-specific sexual behavior and 59% (or 61%) in a population with a gender-similar age-specific sexual behavior. For women who were vaccinated at 25 years of age, the corresponding RAV 10 (or 20) years after vaccination was 26% (or 28%) in a population with a traditional sexual behavior and 16% (or 19%) in a population with gender-similar sexual behavior. Results include indirect protection (herd immunity) from HPV16 infection in nonvaccinated women, as shown for vaccination without catch-up.

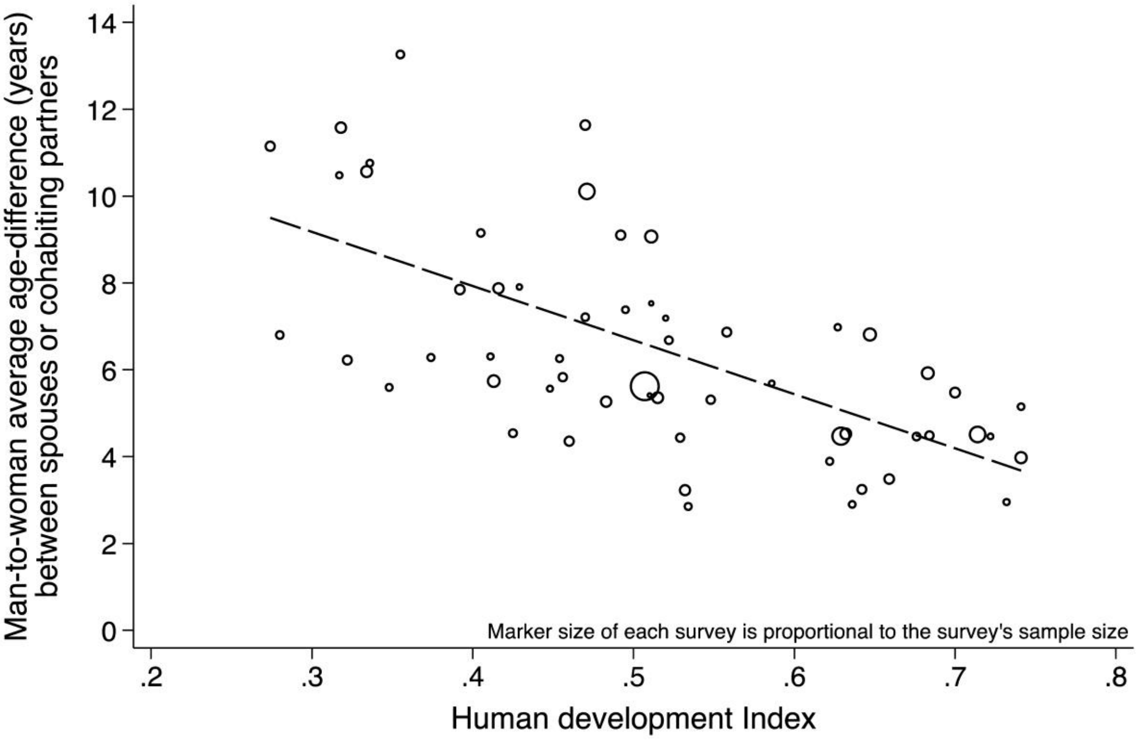

Technical Appendix Figure 1. International correlation between average age difference (from man's age to woman's age) of spouses or cohabiting partners and the Human Development Index (HDI). Each circle is a country represented in a Demographic and Health Survey (*1*) and in the HDI (*2*). The average age difference between spouses or cohabiting partners ranged from 15 years in Burkina Faso to 2 years in Australia (specific countries not shown). The size of each circle is proportional to the size of the DHS survey. Age difference significantly decreases with improvements in a country's HDI: man-to-woman age difference between spouses decreases linearly by –1.25 years (95% CI –16.5 to –8.4) for each 0.1 increase in the HDI (p value = 0.000).

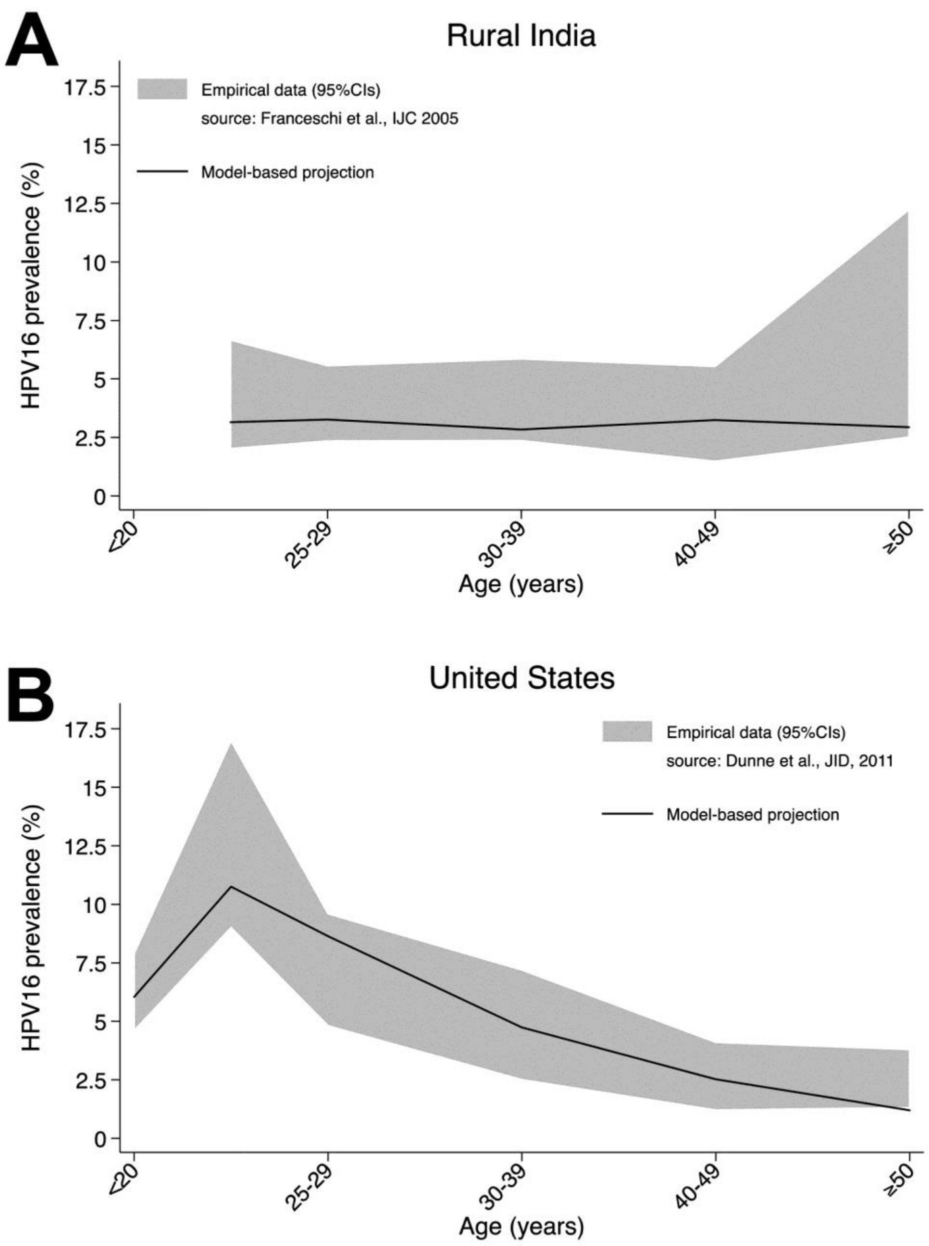

Technical Appendix Figure 2. Comparison of model-based projections and HPV16 prevalence by age group in rural India (*9*) (panel A) and the United States (*10*) (panel B). Model projections were obtained by running the model with the parameters listed in Table 1 of main article. Assumed values were kept constant for both rural India and the United States, whereas calibrated values were specific for rural India and the United States (corresponding to heterosexual populations with traditional and gender-similar age-specific sexual behavior, respectively).

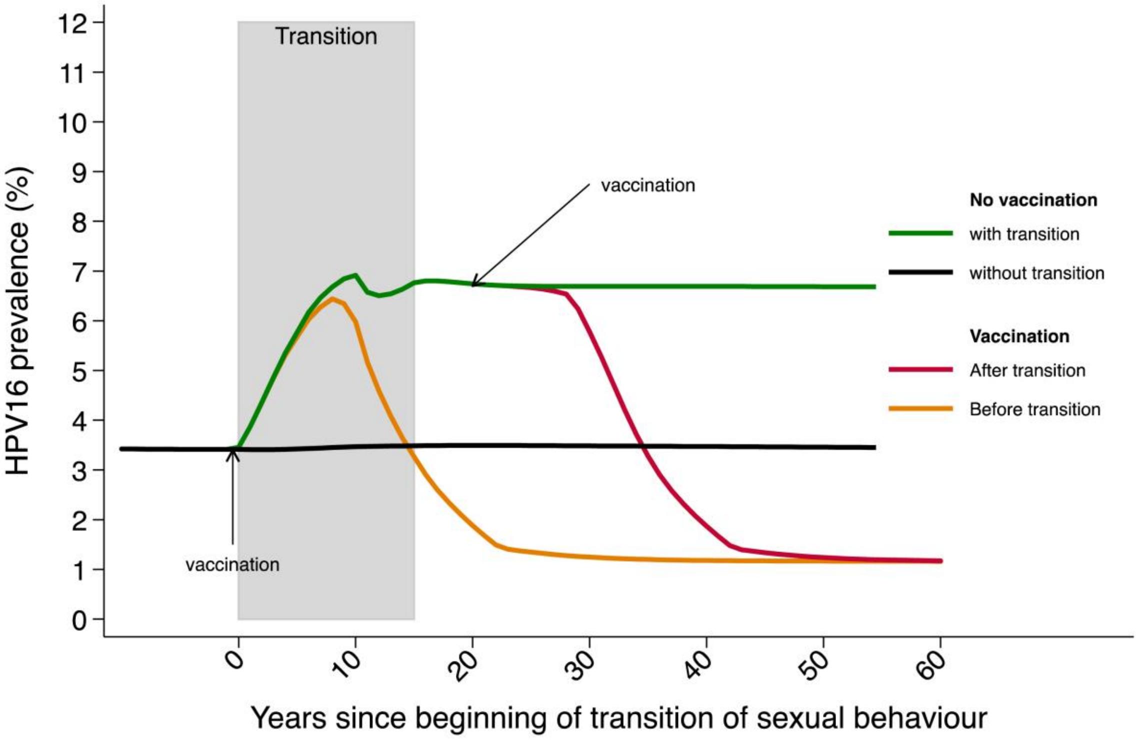

Technical Appendix Figure 3. Model-based age-specific HPV16 prevalence among women 20–34 years of age by population type (i.e., whether a traditional population with a considerable age gap between spouses or a population with similar ages between spouses or cohabitating partners) and changes in HPV16 prevalence in relation to timing of transition in age-specific sexual behavior. For this sensitivity analysis, we changed the value of assortative mixing by sexual activity from 0.7, the calibrated value, to 0.3 (on a scale where fully and randomly assortative mixing correspond to values 0 and 1, respectively).

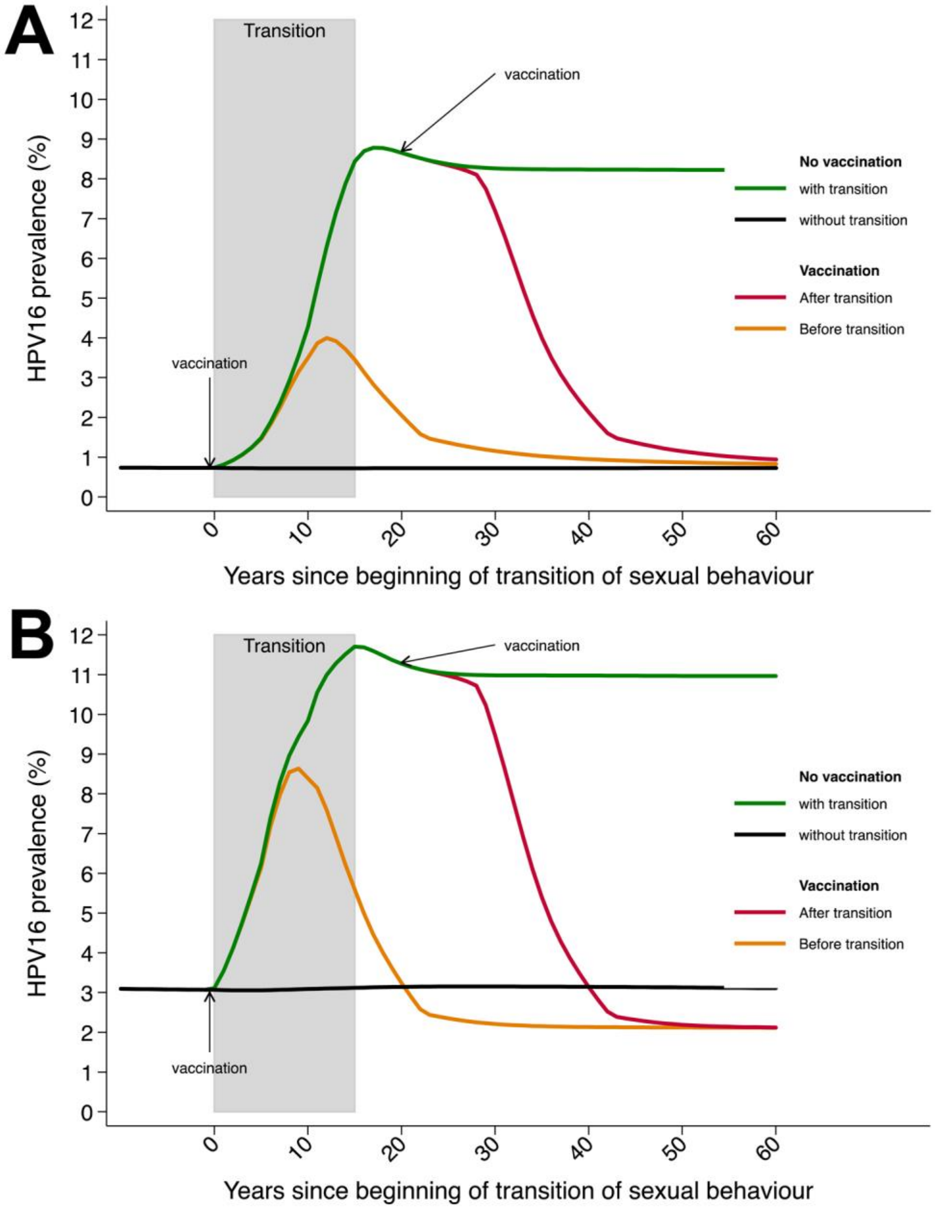

Technical Appendix Figure 4. Model-based age-specific HPV16 prevalence among women 20–34 years of age by vaccination status, population type (i.e. traditional population with a considerable age gap between spouses and a population with similar ages between spouses or cohabitating partners), and years since a traditional population transitioned (hypothetically) to a gender-similar population, assumptions previously described (*12*). Assumed average annual number of partners for both populations was 1.5 (panel A) and 2.0 (panel B).
